# Diversity of Experience in Dental Practice

**Published:** 1874-10

**Authors:** 


### Editorial.

### DIVERSITY OF EXPERIENCE IN DENTAL PRACTICE.

It can hardly have escaped the notice of any that, in reporting upon and describing modes and processes in dental practice, very great variety in experience is exhibited. One finds a certain operation or method usually or, perhaps, always successful, resulting in just the object sought.

The experience of another in the same matter has been very unsatisfactory and failure the usual result. This diversity is found with men of large experience and recognized ability. To such an extent does this exist that those of less experience and age in the profession, often find themselves in uncertainty as to the proper course to pursue. They see one practitioner, of intelligence and long experience, advocating or recommending one method, while another with apparently as much intelligence and experience insists upon another and perhaps a a very different mode or process. Now it may be, as is very often the case, that each is entitled to respect and credence, except when extra enthusiasm carries beyond a reasonable point. Ordinarily the inexperienced will fall in with the views and adopt the practice of the one or the other without attempting to investigate and understand the cause of the diverse opinions and practice. Usually the solution of the matter in such cases will be found in some or all of the following circumstances. Early education or bias has much to do with all the future courses of life, its strength and force will often be modified by inate peculiarities. The man of strong will, and disposed to follow a routine course, will hold on to old methods and maintain his early fixed position with tenacity. The course of instruction in the dental profession up to comparatively a recent period has been fragmentary and without anything like system or uniformity.

He who originates an idea, method or appliance usually regards it as his special prerogative to defend and sustain it against all criticism; and the tenacity with which he does so, generally corresponds to the closeness of his relationship with the point in question. Such an one finds in some a seeming indifference, to what appears to him of so much importance; in others, he finds opposition more or less defined and energetic in proportion as his idea is in contrast or antagonism to the favorite scheme or plan entertained by them. Occupations, modes, practices or plans oftentimes become favorites with us, because, it may be, of some quality we can not define or make plain to the comprehension of another. The execution of any favorite mode of practice will be productive of better results than any one regarded with disfavor—notwithstanding the latter may be essentially the better of the two. That which we love to do is always most efficiently done. Persistency in purpose, confidence and faith in ability are important elements in the accomplishment of any difficult work.

Variations that occur in dental practice in different localities, are modified by the peculiarities of the respective localities. Susceptibilities exist in some places that are entirely absent in others.

These are some of the conditions from which variations arise. In every instance where such differences are presented, the proper course is to endeavor to ascertain the real ground for diversity, and not rest satisfied by attributing it to mere caprice or whim.

